# CDC Grand Rounds: the Future of Cancer Screening

**Published:** 2015-04-03

**Authors:** Cheryll C. Thomas, Thomas B. Richards, Marcus Plescia, Faye L. Wong, Rachel Ballard, Theodore R. Levin, Bruce N. Calonge, Otis W. Brawley, John Iskander

**Affiliations:** 1 Division of Cancer Prevention and Control, National Center for Chronic Disease Prevention and Health Promotion, CDC; 2 National Cancer Institute; 3 The Permanente Medical Group, Inc., and Kaiser Permanente Medical Center, Walnut Creek and Antioch, California; 4 The Colorado Trust; 5 American Cancer Society; 6 Office of the Associate Director for Science, CDC

Cancer is the second leading cause of death in the United States, with 52% of deaths caused by cancers of the lung and bronchus, female breast, uterine cervix, colon and rectum, oral cavity and pharynx, prostate, and skin (melanoma) (1). In the 1930s, uterine cancer, including cancer of the uterine cervix, was the leading cause of cancer deaths among women in the United States (2). With the advent of the Papanicolaou (Pap) test in the 1950s to detect cellular level changes in the cervix, cervical cancer death rates declined significantly (2). Since this first cancer screening test, others have been developed that detect the presence of cancer through imaging procedures (e.g., mammography, endoscopy, and computed tomography) and laboratory tests (e.g., fecal occult blood tests) (3).

The U.S. Preventive Services Task Force (USPSTF) provides cancer screening recommendations and continually reviews the scientific evidence for the potential benefits and harms of screening (4). USPSTF cancer screening recommendations that are graded A or B (indicating that they are recommended by USPSTF) include those for breast cancer, cervical cancer, colorectal cancer, and for lung cancer in heavy smokers (4) (Table 1); Grade A indicates high certainty that the net benefit is substantial, and Grade B indicates high certainty that the net benefit is moderate, or moderate certainty exists that the net benefit is moderate to substantial. *Healthy People 2020* objectives include cancer-related objectives that address incidence, mortality, and screening for each of these cancers; no objective has been established for lung cancer screening because it was not recommended by USPSTF until 2013, after the *Healthy People 2020* objectives were released (5) (Table 2).

## International Models of Organized Cancer Screening

In the United States, patients frequently receive cancer screening recommendations from a physician during an office visit for a general examination or a medical condition. However, in some parts of the world, such as the Netherlands and the United Kingdom, recommendations for screening are made outside of routine medical care settings. These countries use organized systems to contact all adults for whom screening is recommended to remind them to receive cancer screening at recommended intervals. These systems include comprehensive data collection and evaluation systems that provide feedback to improve quality of screening and minimize breakdowns in the multiple steps of the cancer screening process. In the Netherlands, universal cervical cancer screening every 5 years is available for women aged 35–60 years (6). Even though women in the United States received three to four times more Pap tests than women in the Netherlands, the decreases in cervical cancer deaths during 1970–2010 were similar in both countries (6). In the United Kingdom, a pilot study was conducted that showed approximately 60% of those invited participated in a colorectal cancer screening pilot before full implementation of the Bowel Cancer Screening Programme, which screens adults aged 60–69 years for colorectal cancer every 2 years with guaiac fecal occult blood testing; follow-up colonoscopy is available for persons with abnormal test results (7). In that program, 20 local screening centers are grouped into five program hubs that manage patient screening invitations and recall, process guaiac fecal occult blood tests and their results, and schedule endoscopies with nurses at the screening centers. Although general practitioners in the United Kingdom are not directly involved in conducting the screening program, they receive a copy of the results that are sent to their patients.

## Organized Cancer Screening in a Managed Care Setting

System-level changes that have led to a more organized approach to cancer screening are being implemented in certain health care settings in the United States. Kaiser Permanente Northern California (KPNC) is an example of how a large U.S. managed care plan has organized colorectal cancer screening (8). KPNC patient-oriented interventions to increase colorectal cancer screening include tracking patients aged 51–75 years to monitor their use of screening. Approximately 13,000 fecal immunochemical test kits are mailed per week according to the patient’s birth date (aged 51–75 years) or date of previous screening. Automated reminders and reminder postcards are sent approximately 3 and 6 weeks, respectively, after the initial mailing. KPNC provider-oriented interventions include electronic record-based reminders to providers and tracking patients with a positive fecal immunochemical test to ensure they receive a timely follow-up colonoscopy. Monthly quality assurance reports are sent to each medical center, including information on colonoscopy follow-up for patients with a positive fecal immunochemical test, time to colonoscopy, and statistics on cancer incidence and stage, including detection rates for precancerous lesions. With the support of leadership at all levels of management for this system-level process, KPNC has improved the Healthcare Effectiveness Data and Information Set performance measure for colorectal cancer screening quality from 37% in 2005 to 79% in 2012 in the commercially insured population and from 41% in 2005 to 91% in the Medicare population (9).

## Integration of Primary Care and Public Health

The Affordable Care Act (ACA) has the potential to increase access to Grade A and Grade B preventive health services through increased access to insurance coverage and the elimination of cost-sharing (10). In addition, ACA includes numerous other provisions that could increase the proportion of persons who are screened for cancer, such as provisions related to Medicaid preventive services, patient-centered medical homes, and community health centers (11).

However, even with adequate health insurance, many persons and communities might face substantial barriers to obtaining cancer screening tests. Through the integration of public health and primary care (12), opportunities exist to improve both population and individual health, building on the capacities and extensive networks of clinical and preventive services of well-established public health programs and initiatives. Improvements in cancer control can be achieved through population-based approaches to enhance the use of screening and targeted outreach to populations with higher cancer prevalence.

Public health leaders can coordinate hospitals, managed care plans, and other providers of screening services to develop a community-wide, organized approach to cancer screening (12,13). Examples of core elements include approaches that coordinate and strategically implement the patient- and provider-oriented interventions recommended in the *Guide to Community Preventive Services* (14), such as patient reminders and small media (videos and printed materials), combined with enhanced population-level surveillance of cancer screening measures, ideally through integrated electronic data from health care providers. Public health programs could work with electronic databases maintained by Federally Qualified Health Centers, state Medicaid programs, and private insurers to identify unscreened persons eligible for cancer screening, followed by aggressive outreach to encourage participation in cancer screening. In some communities, public health departments might elect to manage or directly provide population-based preventive screening services to geographically defined, vulnerable populations. State-level health-care reform in Vermont has resulted in the integration of chronic disease management, behavioral health, wellness, and preventive services.

## Opportunities for CDC

CDC’s National Breast and Cervical Cancer Early Detection Program is the only national organized cancer screening program in the United States. For 24 years, this program has provided access to breast and cervical cancer screening services to low income women who have limited or no health insurance. Similar to the organized screening examples already discussed, the National Breast and Cervical Cancer Early Detection Program is built on a public health model that includes a clinical provider network unique to the health care delivery system in each funded state, tribal jurisdiction, or territory. Since the program began in 1991, 4.3 million women have received services, and the program has conducted 10.7 million screening examinations. Approximately 56,600 breast cancers, 152,400 premalignant cervical lesions, and 3,200 cervical cancers were diagnosed during 1991–2011.* Along with an existing network to provide breast and cervical cancer screening to vulnerable communities with limited or no health insurance, this program offers outreach, public education, continuing education for health professionals, quality assurance, and surveillance that can be expanded to accommodate a larger population. For example, the New York State Health Department and its partners are creating the New York State Federally Qualified Health Center Cancer Prevention Registry to provide screening data to local and state organizations to increase screening rates in underserved communities and improve screening services. In addition to providing screening services, CDC’s Colorectal Cancer Control Program emphasizes population-based approaches to increase screening rates across all groups. With this new approach, Colorectal Cancer Control Program grantees are implementing evidence-based strategies in partnership with health care systems, insurers, and others, while also emphasizing the importance of quality assurance in the service provision portion of the program. As ACA increases access to insurance coverage across the nation, collaboration with state Medicaid programs and health care systems, especially those that serve populations with limited or no health insurance or usual source of care, will be important. To advance population-based, organized approaches to cancer screening, systems could be developed so that cancer screening tests are not only recommended when a patient visits a primary care physician for a different medical problem but also are tracked and used to improve cancer screening across communities. In addition, communication and outreach strategies that focus on communities with the greatest need for increased screening are important to improve overall community health measures and address health disparities targeted by CDC programs.

## Summary

Effective cancer screening programs that achieve high screening rates depend on patient, provider, and health care system factors. Although cancer screening participation can be improved by increasing access to primary care services and covering cancer screening tests without out-of-pocket costs for patients, public health leaders might still need to collaborate with the health care systems in their communities to better organize cancer screening at the population level, develop surveillance systems that can accommodate electronic data from multiple providers, and eliminate gaps and disparities in cancer screening participation in vulnerable populations. The lessons learned from successful breast, cervical, and colorectal screening programs in national and international settings might be used in the development of initiatives to further expand cancer screening.

## Figures and Tables

**TABLE 1:** U.S. Preventive Services Task Force Grade A and Grade B cancer screening recommendations, 2014

Cancer type	Recommendation*
Female breast	Grade B: USPSTF recommends biennial mammography screening for women aged 50–74 years.†
Cervical	Grade A: USPSTF recommends screening for cervical cancer in women aged 21–65 years with cytology (Pap test) every 3 years or, for women aged 30–65 years who want to lengthen the screening interval, screening with a combination of cytology and human papillomavirus testing every 5 years.§
Colorectal	Grade A: USPSTF recommends screening for colorectal cancer using fecal occult blood testing every year, sigmoidoscopy every 5 years combined with fecal occult blood testing every 3 years, or colonoscopy every 10 years for adults aged 50–75 years. The risks and benefits of these screening methods vary.¶
Lung	Grade B: USPSTF recommends annual screening for lung cancer with low-dose computed tomography for adults aged 55–80 years who have a 30 pack-year smoking history and currently smoke or have quit within the past 15 years. Screening should be discontinued once a person has not smoked for 15 years or develops a health problem that substantially limits life expectancy or the ability or willingness to have curative lung surgery.**

**Abbreviation:** USPSTF = U.S. Preventive Services Task Force.

* Screening recommendations from other organizations that were current when the USPSTF recommendations were released are included in the full USPSTF statement.

† **Source:** US Preventive Services Task Force. Recommendations for primary care practice. Breast cancer: screening. Rockville, MD: US Preventive Services Task Force; 2009. Available at http://www.uspreventiveservicestaskforce.org/Page/Topic/recommendation-summary/breast-cancer-screening.

§ **Source:** US Preventive Services Task Force. Recommendations for primary care practice. Cervical cancer: screening. Rockville, MD: US Preventive Services Task Force; 2012. Available at http://www.uspreventiveservicestaskforce.org/Page/Topic/recommendation-summary/cervical-cancer-screening.

¶ **Source:** US Preventive Services Task Force. Recommendations for primary care practice. Colon cancer: screening. Rockville, MD: US Preventive Services Task Force; 2008. Available at http://www.uspreventiveservicestaskforce.org/Page/Topic/recommendation-summary/colorectal-cancer-screening.

** **Source:** US Preventive Services Task Force. Recommendations for primary care practice. Lung cancer: screening. Rockville, MD: US Preventive Services Task Force; 2013. Available at http://www.uspreventiveservicestaskforce.org/Page/Topic/recommendation-summary/lung-cancer-screening.

**TABLE 2:** *Healthy People 2020* objectives for breast, cervical, colorectal, and lung cancer incidence, mortality, and screening

Objective	Baseline	Most current data (year)	Target
C-2: Reduce the lung cancer death rate	50.6 per 100,000 population	46.0 per 100,000 population (2011)	45.5 per 100,000 population
C-3: Reduce the female breast cancer death rate	23.0 per 100,000 population	21.6 per 100,000 population (2011)	20.7 per 100,000 population
C-4: Reduce the death rate from cancer of the uterine cervix	2.4 per 100,000 population	2.3 per 100,000 population (2011)	2.2 per 100,000 population
C-5: Reduce the colorectal cancer death rate	17.1 per 100,000 population	15.4 per 100,000 population (2011)	14.5 per 100,000 population
C-9: Reduce invasive colorectal cancer	48.9 per 100,000 population	43.7 per 100,000 population (2010)	41.6 per 100,000 population
C-10: Reduce invasive uterine cervical cancer	8.3 per 100,000 population	7.7 per 100,000 population (2010)	7.5 per 100,000 population
C-11: Reduce late-stage female breast cancer	40.9 per 100,000 population	39.2 per 100,000 population (2010)	38.9 per 100,000 population
C-15: Increase the proportion of women who receive a cervical cancer screening based on the most recent guidelines	84.5%	80.7% (2013)	93.0%
C-16: Increase the proportion of adults who receive a colorectal cancer screening based on the most recent guidelines	52.1%	58.2% (2013)	70.5%
C-15: Increase the proportion of women who receive a breast cancer screening based on the most recent guidelines	73.7%	72.6% (2013)	81.1%

**Source:** US Department of Health and Human Services. Healthy People 2020 topics and objectives: cancer. Washington, DC: US Department of Health and Human Services; 2015. Available at http://healthypeople.gov/2020/TopicsObjectives2020/objectiveslist.aspx?topicId=5.

## Footnotes

* These data were current at the time the Public Health Grand Rounds was presented. More current data are available at http://www.cdc.gov/cancer/nbccedp/data/summaries/national_aggregate.htm.
